# Supplementary material for: Role of Tonsillar Chronic Inflammation and Commensal Bacteria in the Pathogenesis of Pediatric OSA
Source: Front Immunol. 2021 Apr 29;12:648064. doi: 10.3389/fimmu.2021.648064 (PMC8116894; doi:10.3389/fimmu.2021.648064)
Supplement: Supplementary file 1 [file DataSheet_1.zip › Supplementary material/Supllementary Figure legends.pdf]

Supplementary Methods

B cell sorting. B cells were isolated from TMC using the B-cell isolation kit II according to the instructions of the manufacturer (Miltenyi, Bergisch Gladbach, Germany. Purified cell populations used for experiments were >95% (Suppl. Fig. 5).

Supplementary Figure Legends

Supplementary Figure 1. Complete sequence of gating performed for data in Figure 1. A representative cultured sample was chosen to show the full gating process. Singlets were gated by plotting FSC-H vs FSC-A for each sample. Within the singlets population, dead cells were determined by a fixable viability dye. Within the viable gate, lymphoid gate was determined through SSC-A vs FSC-A. Within the lymphoid gate, CD20 vs CD3 contour plots.

Supplementary Figure 2. Putative contaminants of the CD20^{down} cell population. A) Freshly isolated TMC were cultured on IL 2+IL 4 alone or CpG+CD40L+IL 2+IL4, for the time points indicated on the top of each panel. Samples were subsequently analyzed by FACS. Gating strategy is partially illustrated, dead cells were determined by a fixable viability dye (previous gates shown in Suppl. Fig. 1). Cells were stained for surface CD20, CD3, CD11c, CD11b, HLA DR. A) P4 denotes CD20$^{+/down}$ cell population. P5 denotes CD11c^{+} within the CD20$^{+/down}$ population. Lower panel: HLA DR^{+} cells were determined within the CD20$^{+/down}$ CD11c^{+} population. B) P4 denotes CD20$^{+/down}$ cell population. In this case, P5 denotes CD20$^{+/down}$CD11b^{-} as we did not detect any CD11b^{+} cell population in any condition of culture. Lower panel: HLA DR^{+} cells were determined within the CD20$^{+/down}$ cell population.

Supplementary Figure 3. Tonsillar innate lymphoid cells (ILC). Identification of ILC by FACS. Singlets were gated by plotting FSC-H vs FSC-A (P1). Within the singlets population, dead cells were determined by a fixable viability dye (P2). Within the viable gate, lymphoid gate was determined through SSC-A vs FSC-A (P3). Cells were stained for surface CD20 and CD3 on the same fluorophore, CD45, CD94, CD127, CD161, CD56, CD117 and CRTH2. P5 denotes the CD20^{-} and CD3^{-} CD45^{+} cells. Within P5, NK cells and the rest of ILCs were differentiated by CD94 vs CD127. Within the ILC compartment, the different subsets were discriminated by CD117 vs CRTH2 (lower panel).

Supplementary Figure 4. Sequence of gating performed to score the median fluorescence intensity (MFI) for TNFα. Singlets were gated by plotting FSC-H vs FSC-A (P1). Within the singlets population, dead cells were determined by a fixable viability dye (P2). Within the viable gate, lymphoid gate was determined through SSC-A vs FSC-A (P3). Within the lymphoid gate, CD20 (red) vs CD3 (blue) were determined. Last panel: overlaid histograms for TNFα fluorescence from CD20 and CD3 populations. Colored dashed lines indicate the respective value scored to perform Fig. 1D.

Supplementary Figure 5. TNF expression by sorted B cells. Sorted B cells were cultured on IL 2+IL 4 alone or CpG+CD40L+IL 2+IL 4, for the time points indicated on the top of each panel either. Cells were stained for surface CD20, CD3 and intracellular TNF✔. Samples were subsequently analyzed by FACS. Gating strategy is partially illustrated. Singlets were gated by plotting FSC-H vs FSC-A for each sample (upper panels). Within the singlets population, dead cells were determined by a fixable viability dye. Within the viable gate, lymphoid gate was determined through SSC-A vs FSC-A (not shown). CD20$^{+/down}$ gate was determined from the CD20 vs CD3 pseudo-color dot plots. TNF^{+} population was identified by plotting CD20 vs TNF^{+} within the CD20$^{+/down}$ gate.

Supplementary Figure 6. Isotype controls for Figure 3 single antibody (immunohistology). Scale bar, 100 μm. Samples were examined with a Nikon Eclipse Ti-E microscope. A), B), C) respective controls as indicated on top.

Supplementary Figure 7. Isotype controls for Figure 3 full staining with isotype control (immunohistology). Scale bar, 100 μm. Samples were examined with a Nikon Eclipse Ti-E microscope. A), B), C) respective controls as indicated on top.
