# Supplementary figures and images for: Role of Tonsillar Chronic Inflammation and Commensal Bacteria in the Pathogenesis of Pediatric OSA
Source: Front Immunol. 2021 Apr 29;12:648064. doi: 10.3389/fimmu.2021.648064 (PMC8116894; doi:10.3389/fimmu.2021.648064)

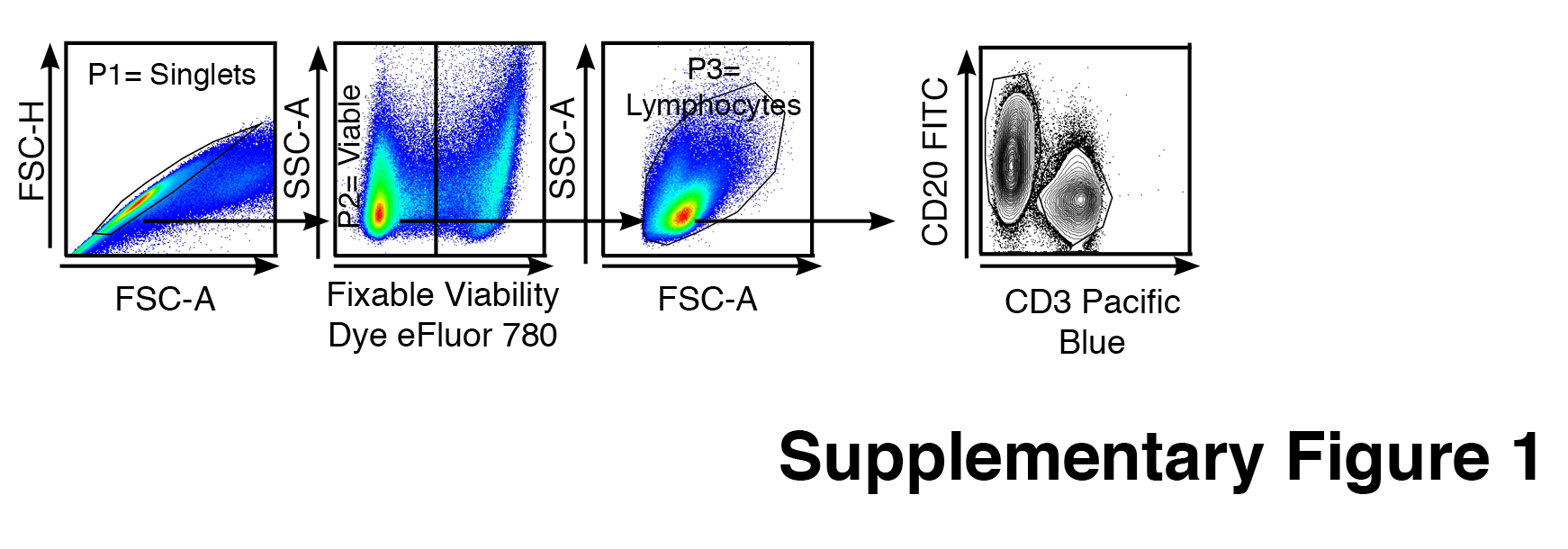

Supplement: Supplementary file 1 [file DataSheet_1.zip › Supplementary material/Suppl Fig 1.tif]

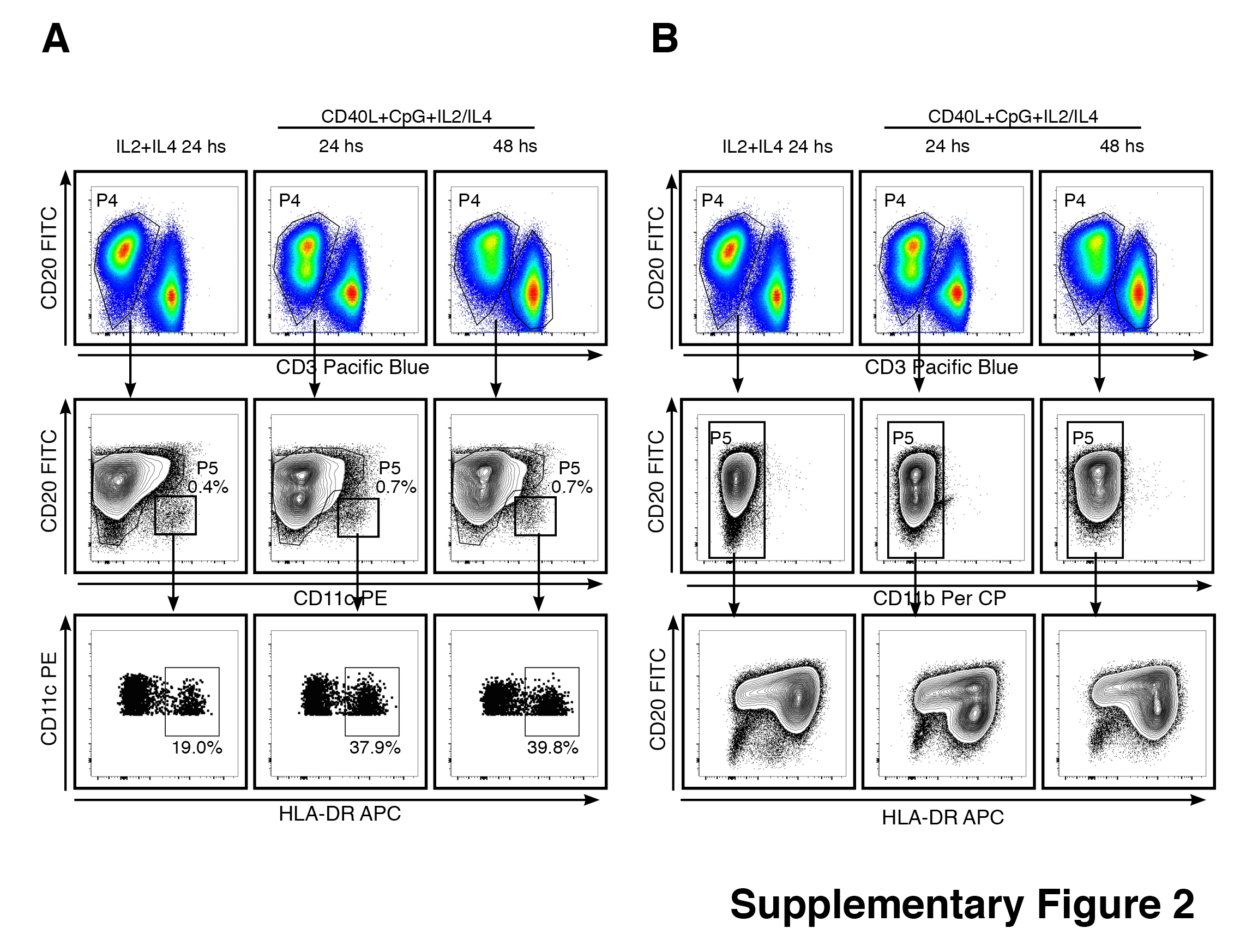

Supplement: Supplementary file 1 [file DataSheet_1.zip › Supplementary material/Suppl Fig 2.tif]

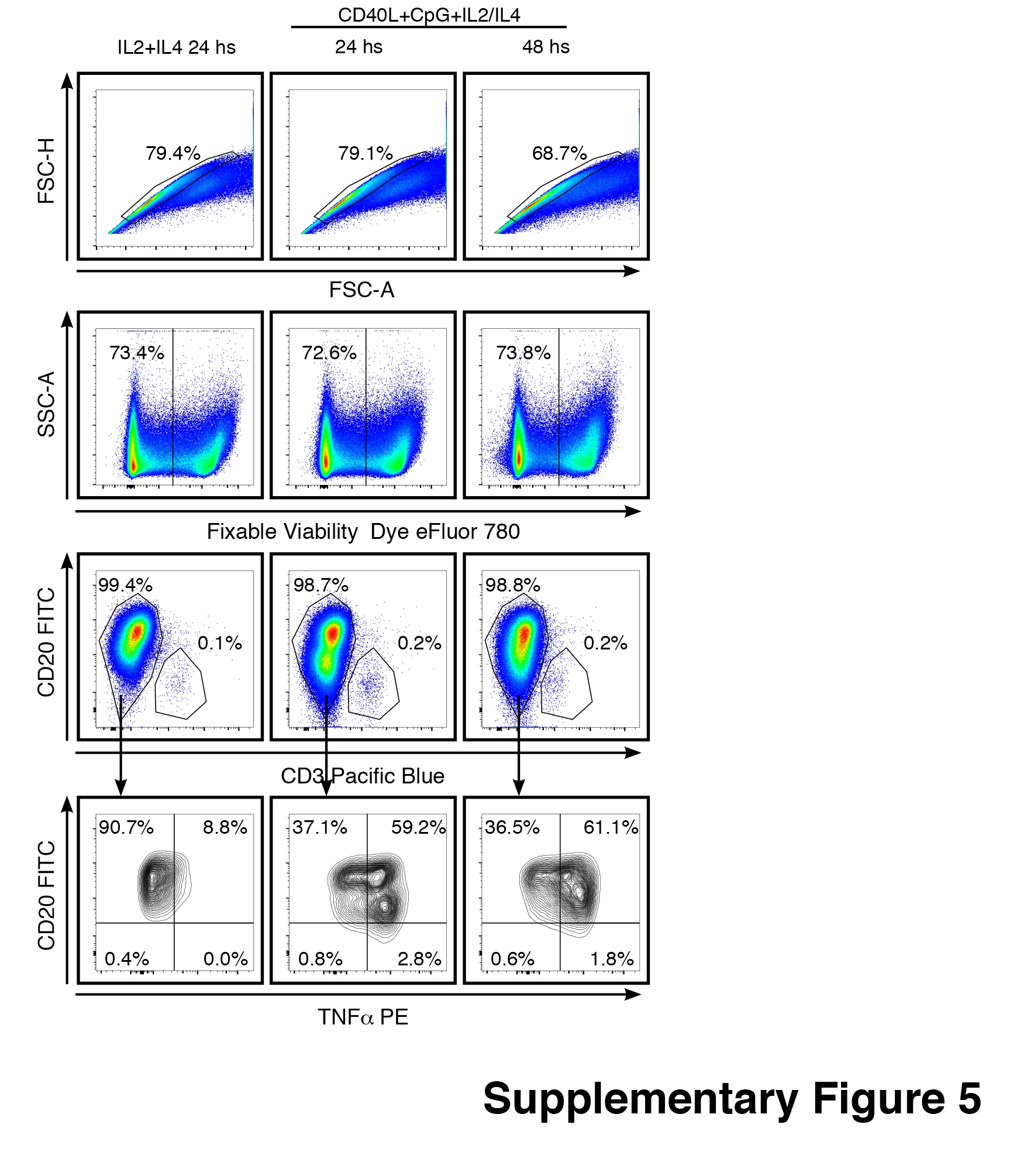

Supplement: Supplementary file 1 [file DataSheet_1.zip › Supplementary material/Suppl Fig 5.tif]

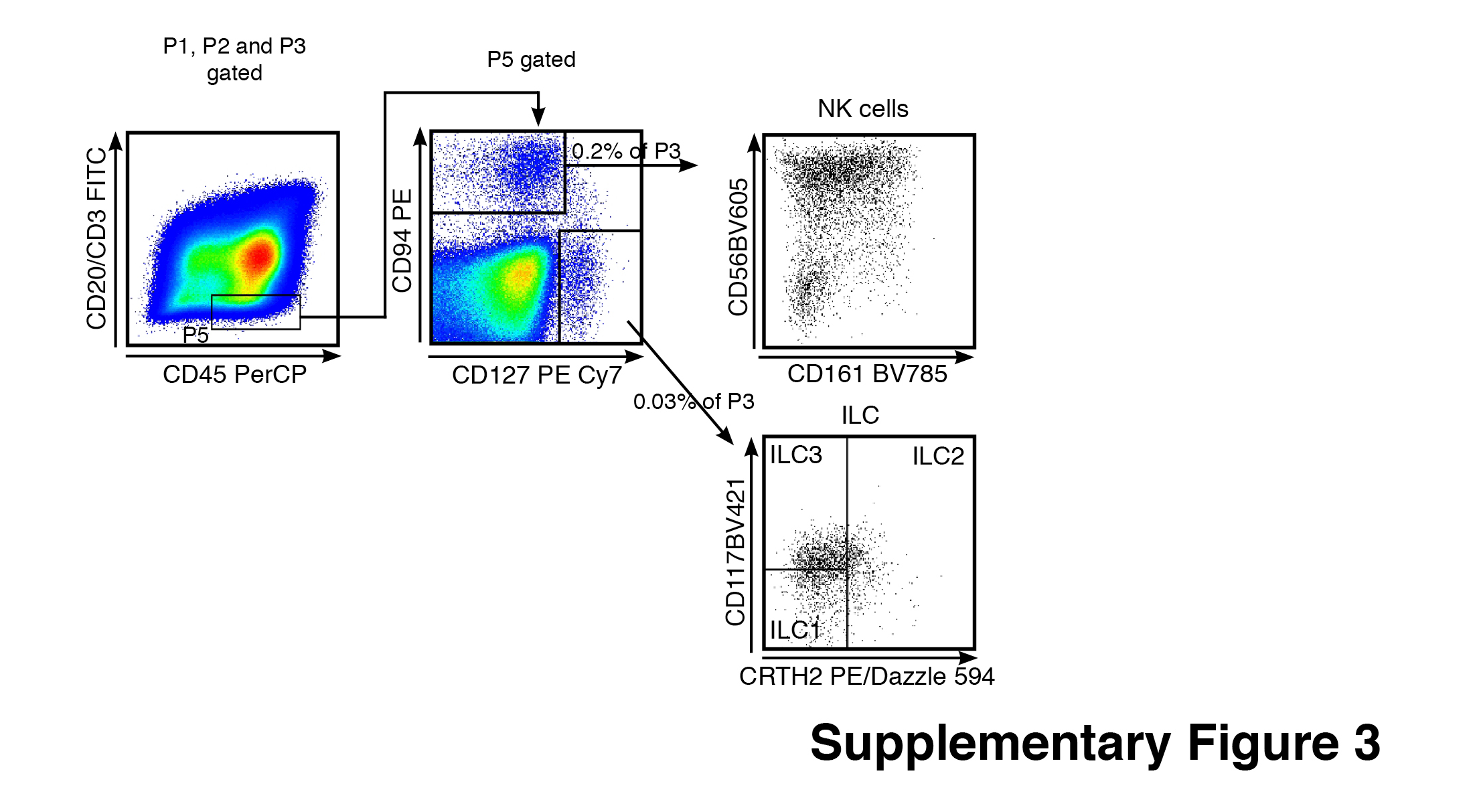

Supplement: Supplementary file 1 [file DataSheet_1.zip › Supplementary material/Suppl Fig3.tif]

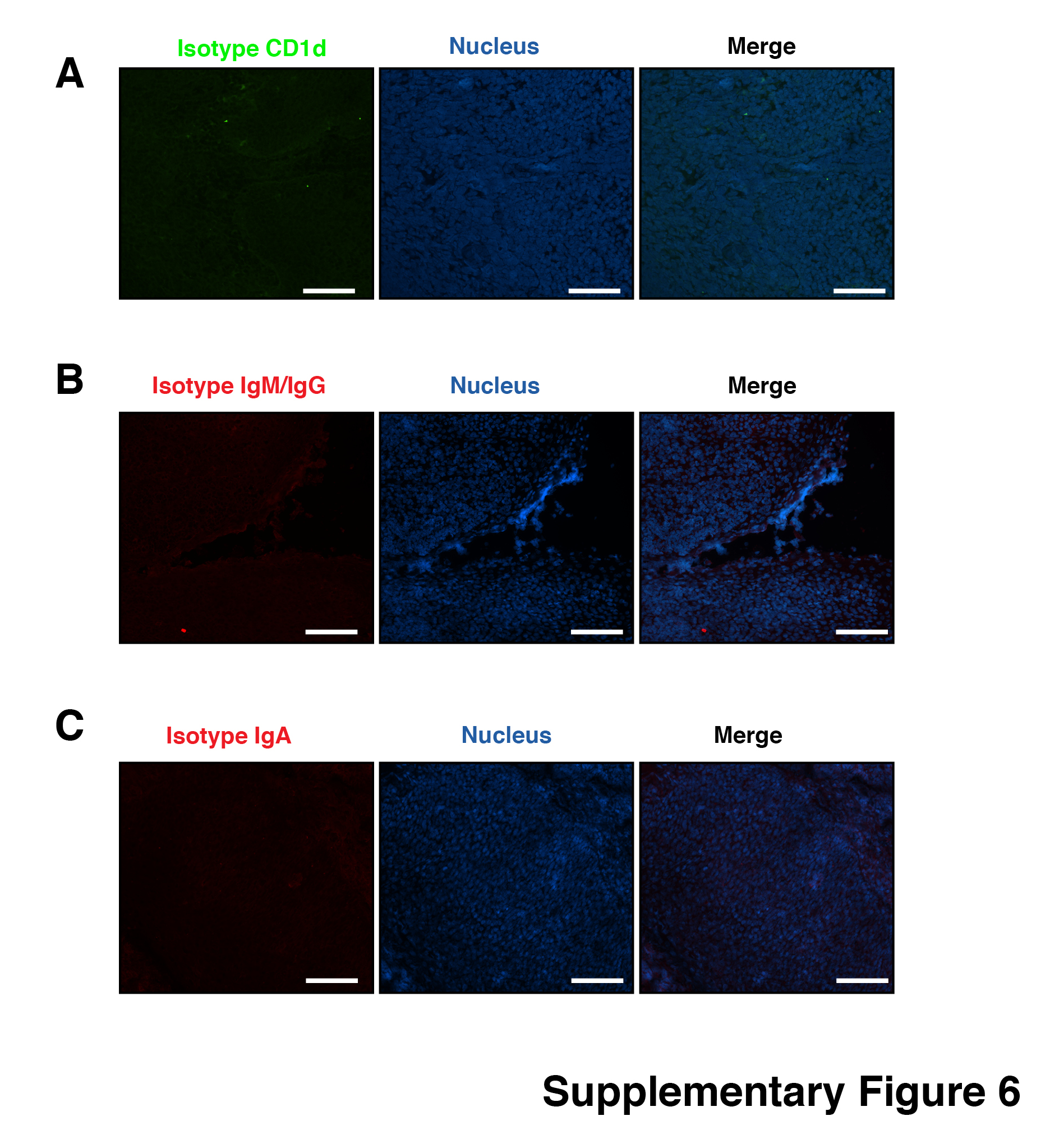

Supplement: Supplementary file 1 [file DataSheet_1.zip › Supplementary material/Suppl Figure 6.tif]

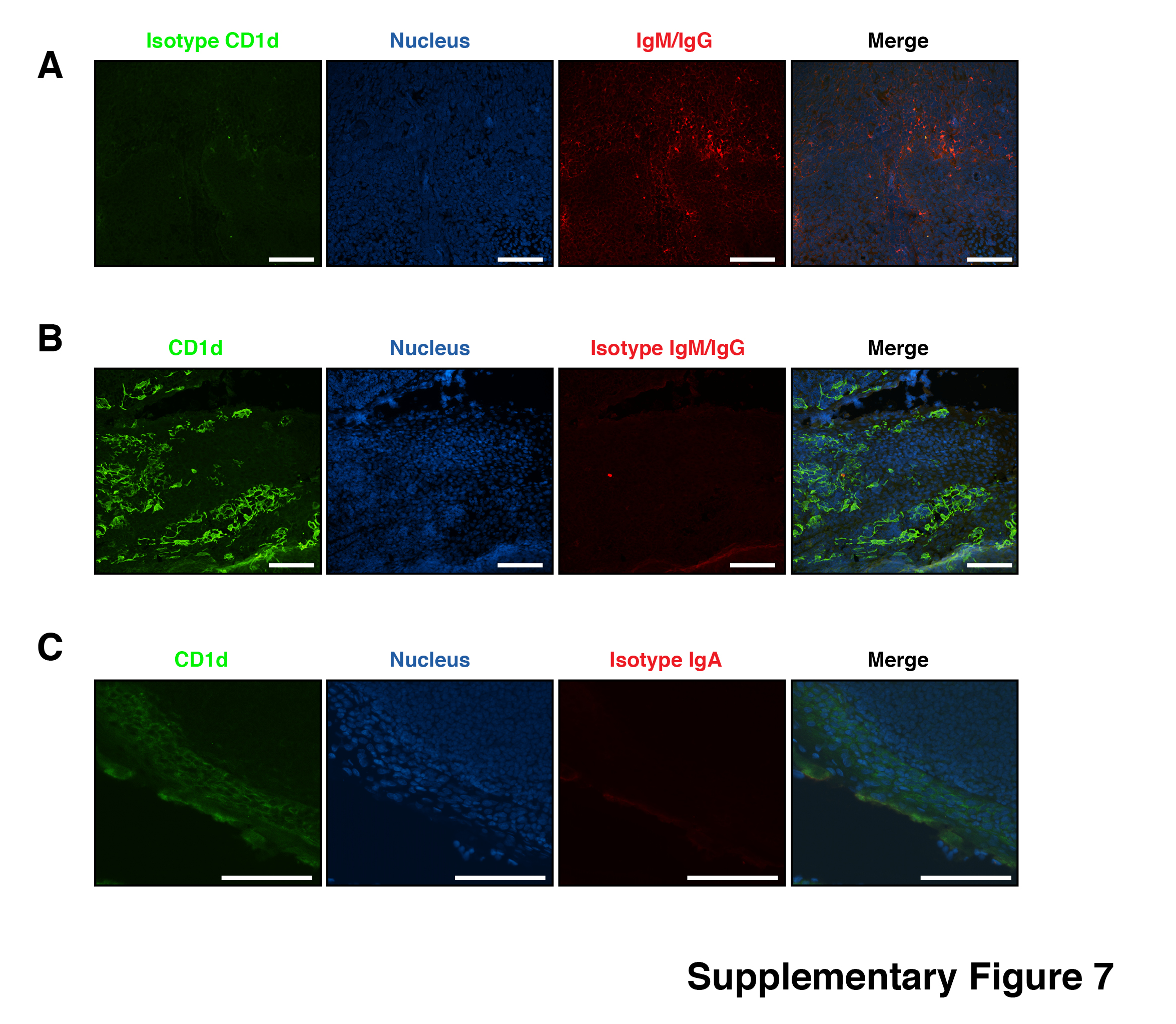

Supplement: Supplementary file 1 [file DataSheet_1.zip › Supplementary material/Suppl Figure 7.tif]

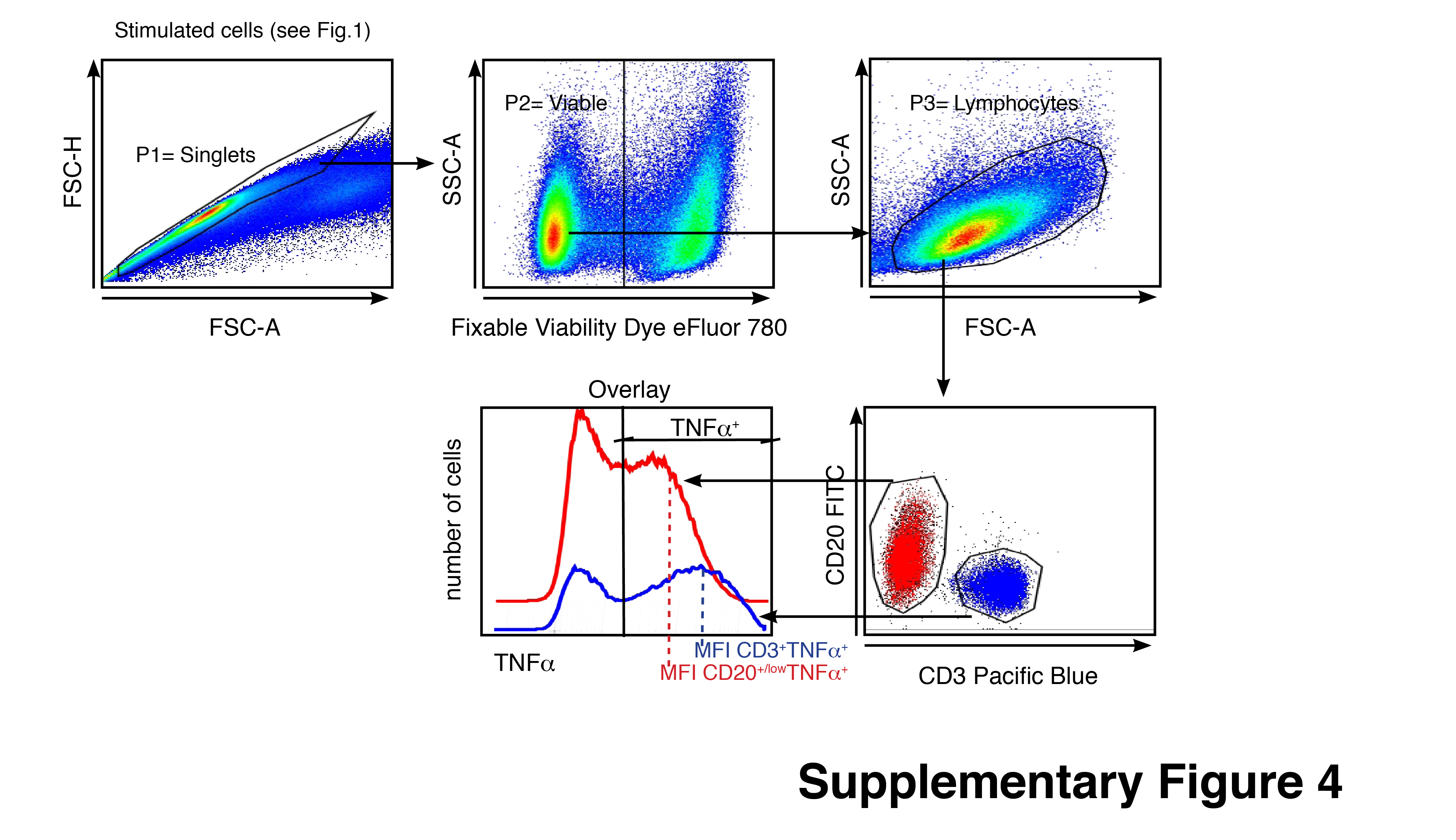

Supplement: Supplementary file 1 [file DataSheet_1.zip › Supplementary material/Supplementary Figure 4 copy.tif]
